# Hyperpolarized Xenon Nuclear Magnetic Resonance (NMR) of Building Stone Materials

**DOI:** 10.3390/ma5091722

**Published:** 2012-09-24

**Authors:** Michele Mauri, Roberto Simonutti

**Affiliations:** Department of Materials Science, University of Milano-Bicocca, Via R. Cozzi 53, 20125 Milano, Italy; E-Mail: michele.mauri@mater.unimib.it

**Keywords:** ^129^Xe NMR, hyperpolarization, continuous flow, adsorption, diffusion, petrography, rocks, weathering, porosity

## Abstract

We have investigated several building stone materials, including minerals and rocks, using continuous flow hyperpolarized xenon (CF-HP) NMR spectroscopy to probe the surface composition and porosity. Chemical shift and line width values are consistent with petrographic information. Rare upfield shifts were measured and attributed to the presence of transition metal cations on the surface. The evolution of freshly cleaved rocks exposed to the atmosphere was also characterized. The CF-HP ^129^Xe NMR technique is non-destructive and it could complement currently used techniques, like porosimetry and microscopy, providing additional information on the chemical nature of the rock surface and its evolution.

## 1. Introduction

The development of optically pumped xenon nuclear magnetic resonance (NMR) that exploits the diffusion of highly nuclear spin-polarized xenon gas on the surface or through samples [1,2] initiated completely new fields of application for the established ^129^Xe NMR: from hyperpolarized biosensors [3] to lung imaging [4], from combustion studies [5] to the characterization of single crystal inner surfaces [6,7,8] and polymer morphology [9]. However, naturally occurring porous systems like stones and rocks are still scarcely studied by continuous flow hyperpolarized xenon (CF-HP) ^129^Xe NMR, except a few cases regarding the characterization of permeability and porosity of reservoir rocks where proof of principle was provided by Mair and coworkers [10]. ^129^Xe is a non-destructive NMR probe for materials due to its virtual lack of chemical reactivity, high isotopic abundance, favorable adsorption properties and high electron polarisability, giving rise to a chemical shift which is strongly sensitive to the shape, size and nature of the material explored by the gas. Xenon atoms, having a Van der Waals diameter of 0.44 nm, can enter a wide range of nanosized structures, and the resulting nuclear magnetic resonance spectra provide rich information on the substrate. Since the discovery of spin exchange between optically pumped rubidium vapors and the nuclear spins of xenon in presence of high partial pressure of ancillary gases like helium and nitrogen [11], hyperpolarized ^129^Xe can be consistently prepared in the laboratory. The spin exchange process can be performed in batches or in continuous flow. The resulting polarization is several orders of magnitude greater than thermal polarization at room temperature even in the field of superconducting magnets [12]. The NMR signal is then proportionally enhanced, so that only a few scans are usually needed for a spectral acquisition, even using gas mixtures where the xenon content is less than 5% in continuous flow [13,14,15]. Characterization time of standard samples is then in the range of minutes. In these conditions, experiments requiring the acquisition of multiple spectra like the study of diffusion by pulsed field gradient echo (PFGE) or the study of chemical exchange by 2D NMR can be performed in a manageable amount of time without xenon enrichment [16].

Rocks are complex systems constituted by several minerals and characterized by a porosity ranging from the microscale to the macroscale. Many types of rocks are used as building stones, sometimes very valuable like travertine, granite or marble. They are also a source of minerals for the preparation of cements and ceramics. The detailed study of rocks, petrography, is carried out with several techniques. Microscopy, including light, birefringence and scanning electron microscopy (SEM) techniques, is most informative for mineral determination, sometimes complemented by X-ray diffraction and microanalysis. Porosity, an important feature of several materials, can be measured by mercury intrusion porosimetry. Each technique also has disadvantages: Microscopy requires a long and delicate preparation including slicing and staining, while mercury porosimetry is destructive.

CF-HP ^129^Xe NMR could provide an additional method for a quick, non-destructive characterization of easily prepared samples, collecting information simultaneously on porosity and chemical composition of the sample. The interaction of xenon atoms with a material produces a chemical shift (δ) relative to the Larmor frequency of the free dilute gas. This observable change can be related to properties of the material including porosity and composition. The volume of a single xenon atom is comparable to molecules composed by lighter atoms. One of the most similar, in terms of size and polarity, is CO_2_. In fact sorption behaviors of the two gases were proven to be comparable in several materials, like for example the metal-organic framework MIL-53(Al) [17]. The interaction of CO_2_ with rocks is a relevant phenomenon for chemical weathering and also a possible method for greenhouse gas sequestering by chemical reaction with silicates [18]: A fast characterization of the accessibility could help in predicting reactivity kinetics.

Since the seminal work of Ito [19] on model zeolites, the observed ^129^Xe chemical shift ( $\delta_{obs}$ ) was treated as summation of separate contributions, representing the different interactions that xenon undergoes while in contact with the material. This approach was extended to several other microporous materials including clays and carbon black, and was summarized recently by De Menorval [20] in the form:

(1) $$\delta_{obs}=\delta_{0}+\delta_{S}+\delta_{X}+\delta (Xe-Xe)\rho$$

Where $\delta_{0}$ is the resonance of the free gas, $\delta_{S}$ the chemical shift of the Xe-surface interaction and $\delta_{X}$ the shift caused specifically by the presence of charged ions or paramagnetic centers, *ρ* is the gas density. The last term in the equation accounts for Xe-Xe interactions within the constraints of micropores.

The chemical shift of dilute gas at room temperature is used as reference (*δ* = 0 ppm). The resonance $\delta_{0}$ of the sampling gas depends on temperature and pressure. This effect derives from Xe-Xe collisions in the gas phase and can be calculated independently from the sample present in the spectrometer coil [21].

The $\delta_{S}$ and $\delta_{X}$ contributions describe interactions between xenon and the sample and do not depend on the density of the gas. The underlying assumption is that the environment inside the micropores is uniform on the NMR timescale, and xenon acts as nanometric probe: Atoms move fast enough to explore the microporous phase as a whole and average the interaction with walls and ions, mediating non-equivalent sites.

The $\delta_{S}$ contribution is due to collisions between xenon and solid surfaces during the random walk, without specific interaction. In this case, the shift is described by [22]:

(2) $$\delta_{\text{Xe}}=243\left(\frac{2.054}{2.054+\text{Γ}}\right)$$

where Γ indicates the mean free path between subsequent collisions and can be calculated from the geometric parameters of the confining material.

The $\delta_{X}$ term contains the effects of the electrical field of charged ions and the magnetism of atoms and clusters [23]. In other words, $\delta_{X}$ depends on the chemical nature of the surface and is not an analytical function derived from the geometry of the material. Collisions between xenon and the surface always result in downfield shift, while interaction with certain cations can also result in upfield signals (<0 ppm). This effect was rarely seen experimentally, and only one major example in the literature has been extensively studied: silver-exchanged sodium X zeolites [24,25]. Upfield shift is instead typical of xenon molecular compounds [26]. In absence of proper chemical bonding, the xenon chemical shift appearing in AgX zeolites was attributed by Springuel-Huet and coworkers [27,28] to 4d_π_–5d_π_ electron-donation from Ag^+^ to xenon involving the Ag 4d^10^ and the xenon 5d^0^ orbitals. However, other authors have recently explained the upfield shift on the grounds of Density Functional Theory calculations considering Xe adsorption on small Ag_n_^+^ clusters [29].

Finally, the Xe-Xe interactions within the micropores are treated as linear function of gas density *ρ*, with a parameter δ(Xe−Xe) . Since thermally polarized xenon produces very small signals at low pressure, early works on ^129^Xe NMR required several measurements on sealed NMR tubes with different Xe densities, and back extrapolation to zero pressure in order to isolate the $\delta_{S}$ and $\delta_{X}$ contributions.

Working with hyperpolarized xenon, significant signal intensity can be achieved with very low gas density *ρ*. In this work, as described in the experimental part, we used xenon partial pressure of only 0.02 atm meaning the δ(Xe−Xe)ρ contribution in Equation (1) can be neglected. In these conditions, $\delta_{0}$ is also very close to zero. Thus, CF-HP ^129^Xe NMR spectra only contain the $\delta_{S}$ and $\delta_{X}$ contributions that depend on the geometry and chemical nature of the micropores. Notably, there is no dependence on temperature.

**Figure 1:**
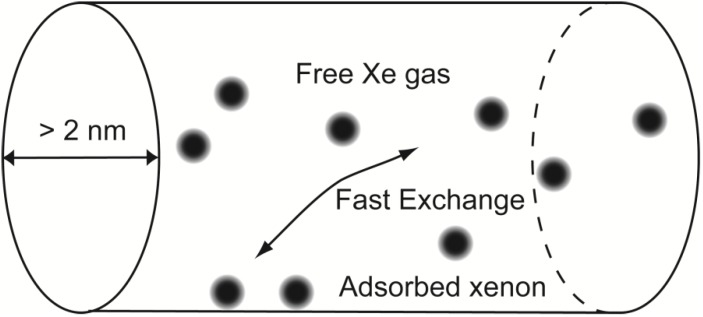
Model of interaction between gaseous xenon and an idealized cylindrical mesopore.

When the pores are, however, in the range of mesopores (2–20 nm diameter, as defined by IUPAC) [30], the mean path of Xe is short in respect of the actual dimension of the pore. The chemical shift is best modeled as an exchange phenomenon between free and adsorbed xenon, as depicted in Figure 1. In other words, pores are large enough that a part of the gas atoms contained in the pore has no interaction with the surface at any given moment. The detected chemical shift is an average between the shift associated with the free gas and the shift of xenon residing on the surface ( $\delta_{S}$ , $\delta_{X}$ ) weighted by the residence time.

The equilibrium condition between bound and free gas can be described in an exchange model as proposed by Terskikh and coworkers [31]. For a mesoporous system composed by cylindrical pores with a volume V and a surface S, the following equation can be defined for vanishing xenon pressures, based on the equation derived by the authors, but considering the surface interaction as the summation of geometrical constraints and charged ion effect:

(3) $$\delta =(\delta_{S}+\delta_{X}){\left(1+\frac{V}{SK_{0}R\sqrt{T}}e^{-\frac{\Delta H_{ads}}{RT}}\right)}^{-1}$$

In this equation, $\Delta H_{ads}$ is the Xe adsorption enthalpy on the surface, while $K_{0}$ is the pre-exponential factor determining the dependence of the Henry coefficient *K* itself upon the temperature. The factor $\delta_{S}$ has been proven to be between 105 and 120 ppm for most silica-based materials that do not contain paramagnetic contributions [31].

Since neither Equation (2) or Equation (3) take into account the Xe-Xe interactions, in given temperature conditions CF-HP ^129^Xe NMR chemical shifts are univocally determined only by the surface chemistry and pore geometry of the material, whether the system is best described as microporous or mesoporous, and whether the shift is positive or negative.

In the present work, CF-HP ^129^Xe NMR was used to investigate natural stones, including marbles, granites, serpentinites and others, producing distinctly different spectra. The different behaviors were explained considering the texture and composition of the stones, and the presence and accessibility of porous systems and paramagnetic centers. As such, ^129^Xe NMR is proposed as fingerprinting for complex rock materials.

## 2. Results and Discussion

### 2.1. Fingerprinting of Minerals and Rocks with CF-HP ^129^Xe NMR

**Figure 2:**
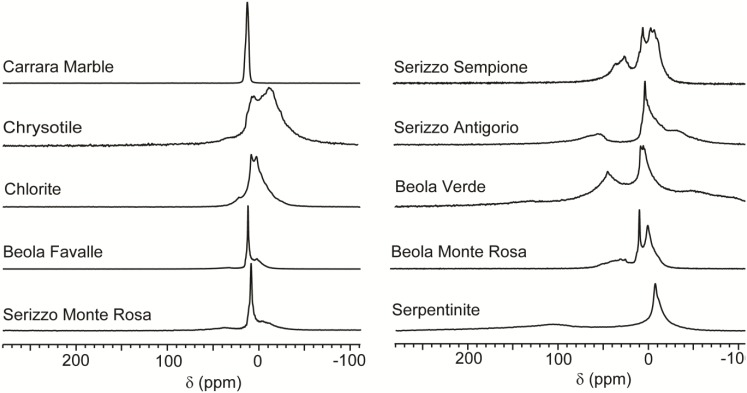
Continuous flow hyperpolarized xenon (CF-HP) ^129^Xe spectra of mineral and rock samples. Signal intensities were normalized arbitrarily to similar height for a more immediate comparison.

Figure 2 shows a selection of the CF-HP ^129^Xe NMR spectra acquired of minerals and rocks. Samples were shaped as regular rectangular cuboids (50 × 6 × 6 mm, see experimental section for more details) in order to fit in the NMR detection coil. Fibrous chrysotile was loosely packed in the sample holder. Once prepared, the cuboids were equilibrated with the atmosphere at room temperature for several days.

The spectra displayed in Figure 2 demonstrate the variety of interactions between stones and xenon gas. Since it has been reported that bulk diamagnetic susceptibility can affect the measured chemical shift of xenon [32] and krypton [33] in the case of macroscopic samples, for each cuboid, several CF-HP ^129^Xe NMR spectra were acquired changing the position and orientation of the cuboid in the sample holder. However, for the materials studied, no differences in the spectra were noticed. Also, spectra acquired from different slices of the same rock provided similar results. Thus each spectrum shown is representative of the material studied. A common feature of the spectra is a peak of varying line width around 0 ppm, but most samples also present a rich variety of signals. Due to the transient nature of the hyperpolarization, and to the low sensitivity of thermally polarized xenon, it is not possible to provide an internal standard in the form of sealed xenon volume. To define a chemical shift reference, a CF-HP ^129^Xe NMR spectrum was recorded during flow of hyperpolarized xenon in an empty sample holder, and the obtained signal of free xenon gas was set to 0 ppm. The signal of free xenon gas in the presence of rocks can be slightly (<10 ppm) shifted from the above-defined zero. This shift does not indicate a specific interaction, but rather the effect of the bulk material on the local magnetic field.

Some samples, exemplified by the Carrara Marble, display only a single peak around 0 ppm with 1 ppm line width or less. This is also the case of Rosso Verona marble (not shown in figure). Carrara Marble is a white veined metamorphic rock with a uniform saccaroid crystalline structure, with crystals ranging from 200 to 600 μm in size and an open porosity of around 2%. The components are mostly dolomite and quartz and other very compact rocks. In this case, the only detected signal is attributed to free xenon flowing around the sample without interaction. Moreover, interactions between the gas and a surface should cause a decrease in T_2_ relaxation time and a concurrent line width increase that was not detected in systems with such low porosity. In fact, all other samples also display a signal around 0 ppm that is broader compared to the cases of Carrara Marble and Rosso Verona. Free xenon gas is always present, since the micro- and mesoporosity of rocks is not sufficient to confine all xenon atoms delivered by the gas flow during the NMR acquisition time, but macroporosity and bulk diamagnetic susceptibility effects [32] produce broader resonances. Several samples present, in addition to free gas resonance, other signals at different chemical shifts whose interpretation needs a detailed analysis of the single stone.

The spectrum of chrysotile has several partially overlapping resonances in the region between 10 and −30 ppm. The line shape is difficult to fit as summation of Gaussian or Lorentzian functions, but at least one peak at −15 ppm is distinguishable from the peak at 0 ppm. Chrysotile is a mineral with a very large surface available for interaction with the gas, since it is formed by a bundle of fibrils (diameter < 1 μm), composed of microfibrils (diameter < 50 nm). Chrysotile is a phyllosilicate with the chemical formula Mg_3_(Si_2_O_5_)(OH)_4_. In real materials, a part of the Mg atoms is replaced by Fe or other atoms. This geometric and compositional complexity explains the presence of several overlapping resonances in the spectrum. As discussed in the introduction, negative chemical shifts for atomic xenon are rarely observed and they are associated with the presence of metal cations like Ag^+^ and Cu^+^ in close contact with the xenon atoms. Chrysotile is a natural material and it contains many metal cations at the ppm level and, among others, Cu^+^. Thus we suggest that copper cations, even though they are highly diluted in respect of the total bulk, are concentrated enough at the fibrils’ surface to provide a measurable effect. This effect is not limited to chrysotile, but also appears in several other samples that contain phyllosilicates, *vide infra*.

The spectrum of chlorite also has different resonances around zero, spanning a spectral region between 10 and −10 ppm. The upfield signal is so close to the gas peak that they partially overlap. Both chlorite and chrysotile also presents a downfield signal appearing as a small peak around 25 ppm. This shift is compatible with the presence of an accessible mesoporous system. In the case of chrysotile, this is possibly due to the empty interfibril spaces rather than adsorption by inherent porosity of the silicate, while in the case of chlorite the effect could be due to grain structure. In both phyllosilicates xenon does not give any large (> 100 ppm) downfield chemical shift signals as seen when the gas explores the interlamellar space of clays [34]. Contrarily to synthetic clays that have been thoroughly emptied and whose galleries are made accessible, the interlamellar space of this sample is inaccessible.

The basic concepts developed in the study of minerals are useful in discussing the spectra of rocks such as different kinds of Beola or Serizzo samples, commercial names that indicate metamorphic gneisses of the Alpine region used as building stones for applications ranging from urban furniture to funeral art. For example, Beola Favalle and Serizzo Monte Rosa are rocks with different macroscopic appearance in terms of texture and color. Their mineralogical composition is instead similar, as indicated in Table 1. They display very similar spectra, where the prevalent signal is the sharp peak attributed to the gas at −5 ppm, indicating a globally low surface interaction. They both display a partially overlapping peak around −15 ppm and a slightly more distant peak at 17 ppm (Beola Favalle) and 28 ppm (Serizzo Monte Rosa). Quartz, plagioclase and potassium-feldspar, the main components of those two rocks, are tectosilicates: very compact minerals without pores accessible to xenon. Upfield and downfield shifts are attributed to biotite or muscovite, phyllosilicates present in both samples.

Biotite is a dark, iron-rich 2:1 mica trioctahedral phyllosilicate with the idealized formula K(Mg,Fe^2+^,Fe^3+^)_3_(AlSi_3_O_10_)(OH)_2_. Biotite has a layered crystal structure and is found worldwide in granites and gneisses [35]. Muscovite is instead a dioctahedral, with composition (KAl_2_(AlSi_3_O_10_)(F,OH)_2_). The occurrence is similar, and the two minerals are often found in the same rock, but muscovite has no iron content and is sometimes labeled “white mica” due to its color. The connection between mineral composition and spectra is demonstrated by ^129^Xe probes of the material at the microscopic level.

Other rocks, including Beola Verde, Beola Monte Rosa, Serizzo Sempione and Serizzo Antigorio display signals both upfield and downfield. The downfield peak is shifted more than 50 ppm and is then attributed to accessible micropore systems. Line width values in the range of tens of ppm are due to a specifically strong dephasing interaction, or simply to diffusion through different adsorption zones [36,37]. An interesting comparison can be made between Serizzo Antigorio and Serizzo Monte Rosa. Serizzo Antigorio has a broad peak at around 60 ppm, while the other sample has no spectral features in that region. The main compositional difference from the Serizzo Monte Rosa is that the biotite fraction is almost double (12% against 7%). A similar comparison could be drawn between Beola Favalle and Beola Monte Rosa. In the latter, the muscovite fraction is around 8%, against the 4% of the former. The resulting CF-HP ^129^Xe spectra suggest that once the percentage of phyllosilicates increases, they are not present as isolated spots but create a complex system of multiple sized pores, resulting in a residence time that is long in the NMR timescale and affects xenon resonance significantly. The spectrum of Beola Monte Rosa has several well-separated peaks that can be identified unambiguously with peak picking algorithms.

This material was then explored in greater detail to elucidate the nature of the shift, the connectivity between pores and the applicability of the models reported in the introduction.

Evaluation of xenon gas transport into the rock was performed by measuring the intensity of the peaks of Beola Monte Rosa as function of a buildup time, defined as the waiting time between a train of 90° saturation pulses, that destroys all the polarization inside the detection coil, and a reading pulse [7,38]. The signal intensity reached a plateau value in about 1000 ms uptake time without significant changes in the line shape, the value is to be compared to 200 ms for the empty sample holder. In order to understand this result, we should consider two limiting situations. Considering the Fickian diffusion in one dimension, like in mesopores, the mean square displacement of xenon can be evaluated by $\langle x{\left(t\right)}^{2}\rangle =2Dt$ where D is the diffusion coefficient. In uptake experiments, we can consider the xenon mean square displacement with a good approximation as the depth of the skin probed by the gas inside the sample. In the limit of macroporosity, xenon travels unhindered through the sample with a diffusion coefficient estimated by Mair in the range of 10^−6^ m^2^/s. In one second, the probed depth is then in the millimeter range. With the given sample shape (six by six section cuboid) the gas saturates a significant percentage of the sample volume prior to acquisition. In the presence of single-file diffusion, the mean square displacement of xenon can be evaluated by $\langle x{\left(t\right)}^{2}\rangle =2F(\theta )\sqrt{t}$ , where *F(θ)* is known as the single-file mobility and *θ* is the fractional channel occupancy; in this case xenon atoms can be hindered further, to values of *F(θ)* as small as 10^−12^ m^2^/s^1/2^ as estimated by Cheng and Bowers [16] for microporous systems with pores sized less than 0.6 nm. In this case, one second buildup times correspond to a probed depth in the micrometer range, reducing the effective fingerprinting region to a minimal skin. Using intermediate values, like 10^−9^ m^2^/s, typical of Xe diffusion in zeolites [39], and not considering single file-diffusion, the probed depth is 30 µm. If this is the case, during the measurement we are probing 2% of the volume of the rocks.

**Figure 3:**
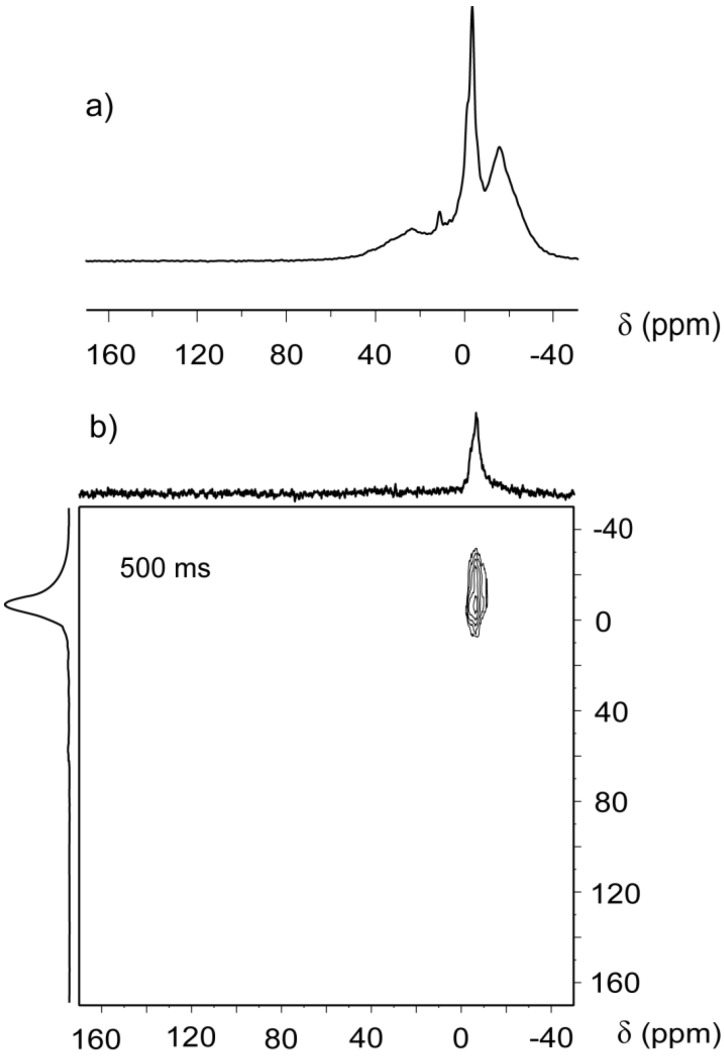
(**a**) CF-HP ^129^Xe spectrum of Beola Monte Rosa; (**b**) two-dimensional continuous flow hyperpolarized (2D EXSY) spectrum acquired with 500 ms exchange time and 1 s recycle delay. F1 and F2 projections are also depicted.

Two-dimensional CF-HP ^129^Xe (EXSY) exchange experiments [9,16] were also performed on the same Beola Rosa cuboid. The 2D spectrum with an exchange time of 500 ms, shown in Figure 3b, contained only an “on diagonal” peak at −6 ppm without the rich spectral features present in the 1D spectrum replotted in Figure 3a. The absence of peaks is due to the flow that continuously purges away the xenon atoms during the mixing period of the EXSY sequence. During the mixing time, xenon atoms do not remain within the pores that generate the −20, 11 and 23 ppm shifts, nor do they have any significant exchange with other pores. Instead, the single signal at −6 ppm is due to a system of macropores with low surface to volume ratio but capable to maintain enough xenon inside the coil for signal detection, at least 500 ms.

Rather than a system of interconnected pores with different sizes, the system is best represented as a combination of several different sized pore systems that are effectively independent and organized in shallow accessible regions close to the surface, in agreement with the buildup measurements.

Further experiments were performed using a liquid nitrogen cooling system to cool both the sample and the incoming flow. CF-HP ^129^Xe NMR spectra were then acquired at intervals of 10 K from room temperature to 220 K.

**Figure 4:**
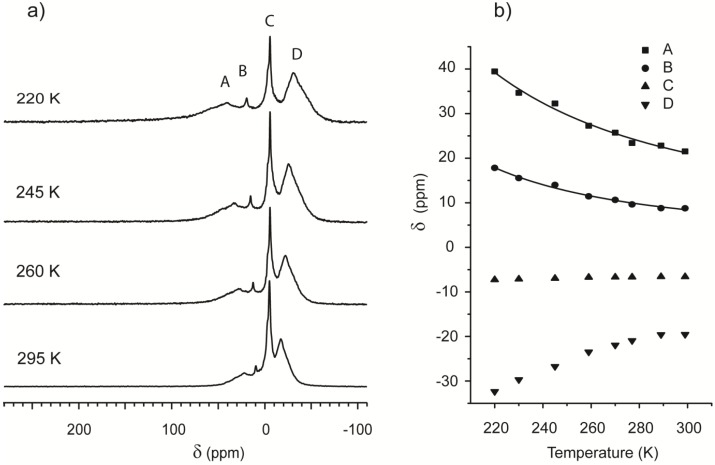
(**a**) ^129^Xe spectra of Beola Monte Rosa at different temperatures; (**b**) ^129^Xe NMR chemical shift for the main resonances present in Beola Monte Rosa spectrum as function of the temperature.

Figure 4 contains a selection of the spectra obtained at different temperatures: Peaks remain well defined and separate in the probed range, indicating several non-interchanging adsorption regions. Peak picking was performed on all the spectra, and the values are plotted in Figure 4b. In the framework of Equation (3), temperature dependence of the shift is a function of the adsorption parameters of each region.

The chemical shift of the peak indicated with the letter C is insensitive to temperature variation. Since it is located at −7 ppm and is the most intense, it is attributed to free gas. A common feature of the other chemical shifts is an apparent convergence toward free gas value at high temperature. Peak D, always being upfield, falls outside the strict definition of Equation (3). The data of the downfield Peaks A and B are instead plotted and fitted using the same equation, confirming that the origin of the chemical shift is consistent with the model presented in Figure 1.

### 2.2. Transient Phenomena on Rock Surfaces as studied by CF-HP ^129^Xe NMR

The experiments described in the previous section were performed on cuboid samples, but the only limitation on sample form is the diameter of the NMR coil where the sample is positioned. Any stone material can be ground to powder and packed loosely in the sample holder. Sample powdering increases surface to volume ratio and should in principle enhance signals in regions associated with porous systems while reducing the relative intensity of the spectral region of free gas. In this way we could obtain more information on samples that are not sufficiently responsive in cuboid shape.

One such sample is serpentinite, a green rock that is made up mostly of antigorite and contains 4% magnetite Fe_3_O_4_. Magnetite, being ferromagnetic, generates a magnetic field that increases the relaxation of the hyperpolarized gas. The lamellar antigorite should favor this interaction by providing accessibility. The CF HP ^129^Xe spectrum shown in Figure 2 presents a broad peak centered at δ = −9 ppm and an even broader spectral feature at around 105 ppm. The peak nearest to zero is typical of free gas interacting with a bulk material, as seen in several spectra in Figure 2. Compared to the multiple signals of Beola Rosa, the spectrum of serpentinite is quite featureless, possibly due to the width of the signal associated to surface interaction (several tens of ppm). To enhance the signal, we ground the serpentinite cuboid to a fine dust using mechanical methods, and then acquired CF HP ^129^Xe NMR on the resulting powder.

**Figure 5:**
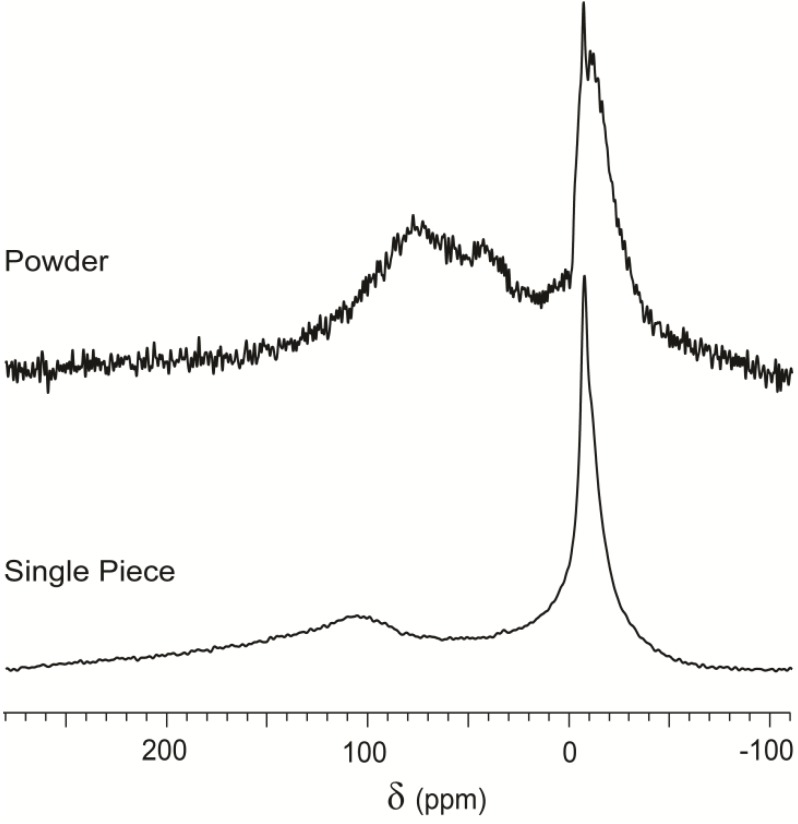
CF-HP ^129^Xe NMR spectra of serpentinite powder and bulk material with the same gas flow and number of scans, normalized to the same maximum intensity of the highest peak.

The spectrum of xenon interacting with a single cuboid of serpentinite is replotted in Figure 5 for comparison with the same material in powder form. Relevant differences appeared in chemical shift, relative intensity of the peaks and total signal intensity. In the powder spectrum, the downfield spectral features (*δ* > 20 ppm) are enhanced relative to the peak associated to free gas, as predicted for an increase of accessible surface. The chemical shift also changed, from a broad peak centered at more than 100 ppm to a complex signal constituted by at least two peaks around 65 and 40 ppm. Signals in Figure 5 have been normalized to the same height for better readability, so the difference in signal intensity appears as significant noise in the spectrum of powdered material. Thus, increased accessible surface produced a decrease of the NMR signal by more than one order of magnitude.

To explain this counterintuitive behavior, we must consider that hyperpolarized Xe magnetization is far removed from the polarization due to the Boltzmann distribution of nuclear spins at thermal equilibrium in a static magnetic field. Thus the high polarization state is transient, and strongly depolarizing interactions with the material during the recycle delay reduce signal intensity. This is usually not an issue due to the amount of xenon flowing in the rf coil continuously, but magnetite is a powerful natural magnetic material, and powdering increases its accessibility. Changes in chemical shift also indicate a qualitative difference between the initial surface and the powder. Since the surface of the single piece was exposed to the atmosphere for several days before the experiment, and the powder was not, we can attribute the spectral differences to the interaction with atmospheric gases. For example, oxygen promotes the conversion of magnetite to hematite, with the reaction:

4 Fe_3_O_4_ + O_2_ = 6 Fe_2_O_3_ (4)

Rocks in the earth crust usually contain redox couples (like magnetite/hematite) whose equilibria are defined by mineral redox buffers, and are determined by local oxygen fugacity. When the rock surface is in contact with the atmosphere, the equilibrium shifts locally towards the most oxidized term.

This general concept is not limited to redox buffers. Paramagnetic centers embedded in the rock can be sensitive to atmospheric oxidation once exposed and can be changed into other, less active, species.

**Figure 6:**
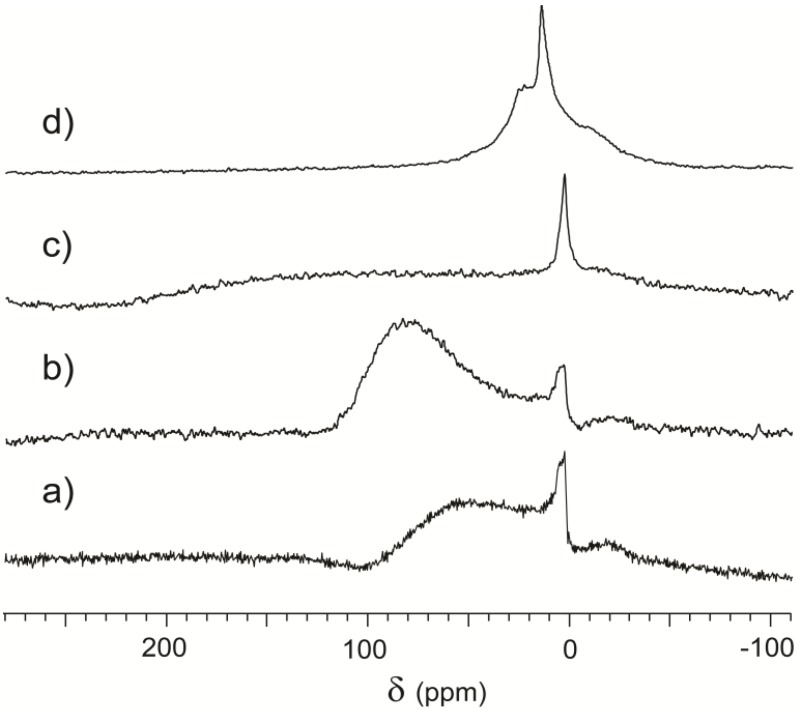
CF-HP ^129^Xe NMR spectra acquired from a Serizzo Monte Rosa sample with different treatments: (**a**) freshly cut sample; (**b**) thirty minutes Xe/ N_2_/He continuous flow; (**c**) four hours contact with the atmosphere; (**d**) twenty-four hours contact with the atmosphere.

For a detailed study of sample evolution over time as function of weathering, we cut a new cuboid-shaped sample from the bulk of a Serizzo Monte Rosa rock. As indicated in Table 1, this particular Serizzo contains more than 10% phyllosilicates (muscovite and biotite), thus providing a variety of local environments with zones of high density and zones of potentially accessible porosity with different chemical compositions. The sample was inserted into the sample holder immediately after the cut to minimize interaction with the atmosphere. The first CF HP ^129^Xe NMR spectrum was acquired as soon as possible after turning on the continuous flow. Other spectra were acquired under different conditions, and details are shown in Figure 6.

In Spectrum 6a, it is possible to separate a peak around 0 ppm, attributed to the gas, and a very broad downfield spectral feature centered around 60 ppm and attributed to the rock surface. Total signal intensity is low, as demonstrated by high noise, similar to the case of powdered serpentinite represented in Figure 5. Spectrum 6b, acquired after 30 minutes of continuous flow, also contains the same two features. The chemical shift of the broader peak increases to around 85 ppm and its intensity also increases. Actually, the integral of the downfield signal far surpasses the integral of the peak around 0 ppm, indicating significant xenon adsorption on the sample.

In between the acquisition of Spectrum 6a and 6b, the sample was immersed into the flow without contact with the atmosphere. The flow contained only xenon and its buffer gases (N_2_, He) and in particular is free of oxygen and water, since it was in contact with extremely reactive Rb vapor during polarization. Rb vapors were in turn thoroughly removed by cooling the gas mixture outside the polarization cell. Such a mixture of inert gases cannot generate chemical modification on the surface. The variation in spectral response of the sample depends on physical sorption and desorption phenomena. The stone-cutting process could not be performed in vacuum, so even the freshly cut sample was in contact with the atmosphere for a few minutes during sample preparation. Thus, pores could be obstructed by atmospheric moisture or other molecules and become inaccessible to xenon atoms. The dry flow promoted desorption of those molecules, thus increasing the accessibility and explaining the differences in intensity between Spectrum 6a and 6b. To interpret the differences in chemical shift, we should mention the Strong Adsorption Site (SAS) model formulated by Fraissard [40] during variable Xe pressure studies of zeolite materials. In some samples, sites of different adsorption strength are present. Strong adsorption implies a large contribution to chemical shift and, concurrently, a long residence time of Xe atoms on the site. In such cases, chemical shift can be very high at low xenon concentration, since the gas is preferentially adsorbed on the SAS. Further increase of xenon concentration actually decreases the chemical shift. Since the SAS are already saturated, excess gas is adsorbed on less interacting sites and rapid averaging between adsorption sites produces a single signal with reduced δ. In our experimental setup, the xenon concentration is kept constant, but there is an effective increase of available sites due to desorption of entrapped molecules like water and oxygen. In those conditions, xenon selectively explores the most interacting sites now available, explaining the increase of chemical shift from Spectrum 6a to 6b.

When the sample was put in contact with the atmosphere for several hours, we obtained spectra where the peak around 0 ppm is prevalent. After four hours of weathering, we also observed a very broad spectral feature spanning hundreds of ppm and difficult to separate from the baseline, plotted in Figure 6c. The exposed material adsorbed atmospheric chemicals to a much greater extent than during sample preparation resulting in a wide array of local environments. Co-adsorption is known to increase the chemical shift [41], and the diffusion of xenon through volumes with highly different interactions causes line broadening. Spectrum 6c then represents a transient state in the evolution of the rock surface, where very high heterogeneity does not produce specific signals.

Finally, after a long equilibration time in air, the sample reverts to being similar to those in Figure 2. This is displayed in Spectrum 6d, where a free gas peak is surrounded by two shoulders, representative of the equilibrium porous structure and the effect of the paramagnetic centers that are not susceptible to atmospheric alteration.

## 3. Experimental Section

**Figure 7:**
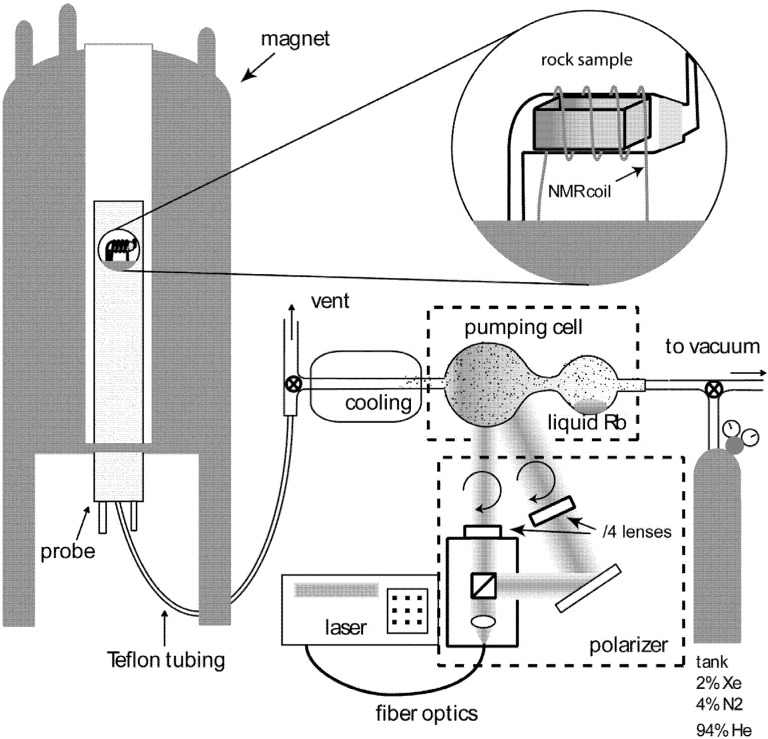
Depiction of CF-HP ^129^Xe apparatus. Components detailed in the text are not represented on the same scale.

The home-built apparatus for CF-HP ^129^Xe is depicted in Figure 7. A system of tanks and pressure regulators provides a fixed pressure of 4 atm (≈ 400 KPa) in the pumping cell. The pumping mixture composition is 2% xenon, 4% nitrogen and 94% helium. A fiber coupled diode array laser (Coherent FAP-System) delivers 16 W at 795 nm. Circular polarization of the light is achieved using a beam splitting cube (Thorlabs), a gold mirror (Thorlabs) and quarter-wave lenses (CVI). The pumping cell is located in the fringe magnetic field (5 mT) of the superconducting wide bore NMR magnet (7.04 T) and contains metallic rubidium, heated to 448K to provide sufficient vapor density. The circularly polarized laser light is focalized to the cell and pumps the Rb electronic levels. In the cell, collisions between Rb and Xe promote an exchange of polarization in favor of the nuclear spin levels of xenon. Hyperpolarized Xe is then allowed to flow at ambient pressure over the sample inside the NMR coil via Teflon tubing (Swagelok). Gas flow rates are optimized to highest signal intensity, typically 300 cm^3^/min. The polarization is higher than 4.5%, with an enhancement factor of more than 7000 over thermally polarized gas, as calculated by comparison with a sealed sample of pure isotopically enriched xenon at 1.5 atm (84.4% ^129^Xe isotope). Therefore, the contribution of thermally polarized Xe is negligible in the experiments, its signal being at least three orders of magnitude smaller than the hyperpolarized one. NMR experiments are carried out on a Bruker Avance 300 spectrometer operating at Larmor frequency of 83.02 MHz for ^129^Xe. Normally spectra were acquired using 1 s of recycle and 512 scans.

Rock samples are cut in single regular large pieces with a cuboid shape and the following dimensions: 50 × 6 × 6 mm. We decided to use this sample geometry since it is easy to achieve and highly reproducible.

Thin slices are prepared and investigated with SEM and EDX microanalysis in order to determine the different minerals composing the rock. Results are summarized in Table 1.

**Table 1:** Microscopic description of rocks used in this paper. Only main components are listed, resulting in a total percentage < 100 for some samples.

Sample	Mineral composition (%)				
Beola Favalle	Quartz (51%)	Plagioclase (18%)	K-Feldspar (19%)	Biotite (8%)	Muscovite (4%)
Beola Monte Rosa	Quartz (52%)	K-Feldspar (13%)	Plagioclase (13%)	Muscovite (8%)	Biotite (4%)
Beola Verde	Plagioclase (54%)	Epidote (16%)	Chlorite (12%)	Muscovite (9%)	Quartz (4%)
Carrara Marble	Calcite (98%)	-	-	-	-
Serizzo Antigorio	Quartz (30%)	Plagioclase (26%)	Orthoclase (25%)	Biotite (10%)	Muscovite (5%)
Serizzo Monte Rosa	Quartz (49%)	Plagioclase (20%)	K-Feldspar (20%)	Biotite (8%)	Muscovite (3%)
Serpentinite	Antigorite (80%)	Olivine (10%)	Diopside (6%)	Magnetite (4%)	-

## 4. Conclusions

In this work, we presented continuous flow HP ^129^Xe NMR spectra of several minerals and rocks. We have shown that ^129^Xe NMR, together with a hyperpolarizing apparatus, can provide qualitative information of interest about natural materials without any pretreatment, even considering the complexity associated with the materials themselves. The spectral response of ^129^Xe in contact with rocks could be qualitatively explained considering the texture of the rocks, including the accessibility and nature of the porosity due for example to the presence of phyllosilicates. Also, several observations of rare negative chemical shift of Xenon in common rocks demonstrate the existence of complex Xe-rock interaction previously observed only in the case of Ag clusters in zeolites but possibly due to other trace metals.

Another relevant feature was the presence and accessibility of magnetic minerals, as well as their state of weathering as a result of contact with the atmosphere. In fact, a diversity of spectra can be obtained from the same sample after different treatments, depending both on the chemical reaction between the surface and atmospheric gases and on reversible phenomena like adsorption and desorption. Consequently, the technique can be used to rapidly characterize rocks and minerals, and to assess the presence of contaminants on the surface of stone materials, including building stones and works of art.

## References

[1] Raftery D., Long H., Meersmann T., Grandinetti P.J., Reven L., Pines A. (1991). High-field NMR of adsorbed xenon polarized by laser pumping. Phys. Rev. Lett..

[2] Navon G., Song Y.Q., Room T., Appelt S., Taylor R.E., Pines A. (1996). Enhancement of solution NMR and MRI with laser-polarized xenon. Science.

[3] Schroeder L., Lowery T.J., Hilty C., Wemmer D.E., Pines A. (2006). Molecular imaging using a targeted magnetic resonance hyperpolarized biosensor. Science.

[4] Mugler J.P., Driehuys B., Brookeman J.R., Cates G.D., Berr S.S., Bryant R.G., Daniel T.M., de Lange E.E., Downs J.H., Erickson C.J. (1997). MR imaging and spectroscopy using hyperpolarized Xe-129 gas: Preliminary human results. Magn. Reson. Med..

[5] Anala S., Pavlovskaya G.E., Pichumani P., Dieken T.J., Olsen M.D., Meersmann T. (2003). *In situ* NMR spectroscopy of combustion. J. Am. Chem. Soc..

[6] Sozzani P., Comotti A., Simonutti R., Meersmann T., Logan J.W., Pines A. (2000). A porous crystalline molecular solid explored by hyperpolarized xenon. Angew. Chem. Int. Ed..

[7] Meersmann T., Logan J.W., Simonutti R., Caldarelli S., Comotti A., Sozzani P., Kaiser L.G., Pines A. (2000). Exploring single-file diffusion in one-dimensional nanochannels by laser-polarized Xe-129 NMR spectroscopy. J. Phys. Chem. A.

[8] Comotti A., Bracco S., Ferretti L., Mauri M., Simonutti R., Sozzani P. (2007). A single-crystal imprints macroscopic orientation on xenon atoms. Chem. Commun..

[9] Simonutti R., Bracco S., Comotti A., Mauri M., Sozzani P. (2006). Continuous flow hyperpolarized Xe-129 NMR for studying porous polymers and blends. Chem. Mater..

[10] Mair R.W., Wong G.P., Hoffmann D., Hurlimann M.D., Patz S., Schwartz L.M., Walsworth R.L. (1999). Probing porous media with gas diffusion NMR. Phys. Rev. Lett..

[11] Happer W., Miron E., Schaefer S., Schreiber D., van Wijngaarden W.A., Zeng X. (1984). Polarization of the nuclear spins of noble-gas atoms by spin exchange with optically pumped alkali-metal atoms. Phys. Rev. A.

[12] Zook A.L., Adhyaru B.B., Bowers C.R. (2002). High capacity production of >65% spin polarized xenon-129 for NMR spectroscopy and imaging. J. Magn. Reson..

[13] Seydoux R., Pines A., Haake M., Reimer J.A. (1999). NMR with a continuously circulating flow of laser-polarized Xe-129. J. Phys. Chem. B.

[14] Brunner E., Seydoux R., Haake M., Pines A., Reimer J.A. (1998). Surface NMR using laser-polarized (129)Xe under magic angle spinning conditions. J. Magn. Reson..

[15] Haake M., Pines A., Reimer J.A., Seydoux R. (1997). Surface-enhanced NMR using continuous-flow laser-polarized xenon. J. Am. Chem. Soc..

[16] Cheng C.-Y., Bowers C.R. (2007). Direct observation of atoms entering and exiting L-Alanyl-L-valine nanotubes by hyperpolarized xenon-129 NMR. J. Am. Chem. Soc..

[17] Springuel-Huet M.A., Nossov A., Adem Z., Guenneau F., Volkringer C., Loiseau T., Ferey G., Gedeon A. (2010). Xe-129 NMR study of the framework flexibility of the porous hybrid MIL-53(Al). J. Am. Chem. Soc..

[18] Schuiling R.D., Krijgsman P. (2006). Enhanced weathering: An effective and cheap tool to sequester CO_2_. Climatic Change.

[19] Ito T., Fraissard J. (1982). Xe-129 NMR-study of xenon adsorbed on Y zeolites. J. Chem. Phys..

[20] Van Miltenburg A., de Menorval L.C., Stocker M. (2011). Characterization of the pore architecture created by alkaline treatment of HMCM-22 using (129)Xe NMR spectroscopy. Catal. Today.

[21] Jameson C.J., Jameson A.K., Cohen S.M. (1973). Temperature and density dependence of the Xe-129 chemical-shift in xenon gas. J. Chem. Phys..

[22] Demarquay J., Fraissard J. (1987). Xe-129 NMR of xenon adsorbed on zeolites-relationship between the chemical shift and the void space. Chem. Phys. Lett..

[23] Sears D.N., Vukovic L., Jameson C.J. (2006). Xe nuclear magnetic resonance line shapes in channels decorated with paramagnetic centers. J. Chem. Phys..

[24] Gedeon A., Burmeister R., Grosse R., Boddenberg B., Fraissard J. (1991). Xe-129 NMR for the study of oxidized and reduced AgX zeolites. Chem. Phys. Lett..

[25] Grosse R., Burmeister R., Boddenberg B., Gedeon A., Fraissard J. (1991). Xe-129 NMR of silver-exchanged X-type and Y-type zeolites. J. Phys. Chem..

[26] Forgeron M.A.M., Wasylishen R.E., Penner G.H. (2004). Investigation of magnetic shielding in xenon difluoride using solid-state NMR Spectroscopy and relativistic density functional theory. J. Phys. Chem. A.

[27] SpringuelHuet M.A., Bonardet J.L., Gedeon A., Fraissard J. (1997). Xe-129 MMR for studying surface heterogeneity: Well-known facts and new findings. Langmuir.

[28] Springuel-Huet M.A., Bonardet J.L., Gedeon A., Fraissard J. (1999). Xe-129 NMR overview of xenon physisorbed in porous solids. Magn. Reson. Chem..

[29] Nguyen H.G., Konya G., Eyring E.M., Hunter D.B., Truong T.N. (2009). Theoretical study on the interaction between xenon and positively charged silver clusters in gas phase and on the (001) chabazite surface. J. Phys. Chem. C.

[30] Rouquerol F.O., Rouquerol J., Sing K.S.W. (1999). Adsorption by Powders and Porous Solids: Principles, Methodology, and Applications.

[31] Terskikh V.V., Moudrakovski I.L., Breeze S.R., Lang S., Ratcliffe C.I., Ripmeester J.A., Sayari A. (2002). A general correlation for the Xe-129 NMR chemical shift-pore size relationship in porous silica-based materials. Langmuir.

[32] Raftery D., Long H., Reven L., Tang P., Pines A. (1992). NMR of optically pumped xenon thin films. Chem. Phys. Lett..

[33] Stupic K.F., Cleveland Z.I., Pavlovskaya G.E., Meersmann T. (2006). Quadrupolar relaxation of hyperpolarized krypton-83 as a probe for surfaces. Solid State Nucl. Magn. Reson..

[34] Sozzani P., Bracco S., Comotti A., Mauri M., Simonutti R., Valsesia P. (2006). Nanoporosity of an organo-clay shown by hyperpolarized xenon and 2D NMR spectroscopy. Chem. Commun..

[35] Nicolini K.P., Lombardi K.C., Schreiner W.H., Mazzaro I., Wypych F., Mangrich A.S. (2009). Evidence of weathering stages of phyllosilicates from biotite/muscovite to kaolinite, probed by EPR spectroscopy. Mineral. Petrol..

[36] Filimonova S., Nossov A., Duemig A., Gedeon A., Koegel-Knabner I., Knicker H. (2011). Evaluating pore structures of soil components with a combination of “conventional” and hyperpolarised (129)Xe NMR studies. Geoderma.

[37] Filimonova S.V., Knicker H., Hausler W., Kogel-Knabner I. (2004). Xe-129 NMR spectroscopy of adsorbed xenon as an approach for the characterisation of soil meso- and microporosity. Geoderma.

[38] Cheng C.-Y., Bowers C.R. (2007). Observation of single-file diffusion in dipeptide nanotubes by continuous-flow hyperpolarized xenon-129 NMR spectroscopy. Chemphyschem.

[39] Heink W., Kaerger J., Pfeifer H., Stallmach F. (1990). Measurement of the intracrystalline self-diffusion of xenon in zeolites by the NMR pulsed field gradient technique. J. Am. Chem. Soc..

[40] Gedeon A., Bonardet J.L., Fraissard J. (1993). Xe-129 NMR study of the CU^+^-Xe interaction in Cu-NaY zeolites-generalization to the demonstration of Xe-*n*d^10^ interactions. J. Phys. Chem..

[41] Gedeon A., Ito T., Fraissard J. (1988). Study of the H_2_O NaY system—An example of the application of Xe-129 NMR of the xenon probe to the investigation of the location of adsorbed phases. Zeolites.

